# RDW-to-ALB Ratio Is an Independent Predictor for 30-Day All-Cause Mortality in Patients with Acute Ischemic Stroke: A Retrospective Analysis from the MIMIC-IV Database

**DOI:** 10.1155/2022/3979213

**Published:** 2022-12-15

**Authors:** Ping Liu, Su Luo, Xiang-jie Duan, Xiang Chen, Quan Zhou, Yan Jiang, Xia Liu

**Affiliations:** ^1^Child Health Care Department, The First People's Hospital of Changde, Changde, China; ^2^Operating Department, The First People's Hospital of Changde, Changde, China; ^3^Infection Department, The First People's Hospital of Changde, Changde, China; ^4^Nursing Department, The First People's Hospital of Changde, Changde, China; ^5^Science and Education Department, The First People's Hospital of Changde, Changde, China; ^6^Oncology Department, The People's Hospital of Shimen County, Changde, China

## Abstract

**Purpose:**

Previous studies have shown that the peripheral red blood cell distribution width (RDW) and human serum albumin (ALB) were both predictors of the risk and mortality of cerebrovascular diseases, and the ratio of RDW to ALB (RAR) was a combined new index that can predict the prognosis of the cardiovascular and respiration systemic diseases, but its role in cerebrovascular diseases had not been effectively evaluated. This study is aimed at exploring whether RAR can effectively predict the 30-day all-cause mortality of acute ischemic stroke (AIS) patients.

**Methods:**

This retrospective cohort study was conducted on AIS patients (age > 18 years) in the intensive care database MIMIC-IV. The RAR was measured based on the red blood cell distribution width and albumin. The main result was 30-day all-cause mortality, and the secondary results were ICU mortality and hospital mortality. Obtain the odds ratio (OR) estimate from the logistic regression model of log-transformed RAR values and mortality. We had used another database for external validation.

**Results:**

A total of 1412 patients were enrolled, with an average age of 68.8 ± 15.9, including 708 (50.1%) males. When log-transformed RAR values were used as a continuous variable, as the values increases, the risk of death increases (30-day all-cause mortality OR = 4.02 (2.21, 7.32) *P* < 0.0001, ICU mortality OR = 3.81 (1.92, 7.54) *P* = 0.0001, and hospital mortality OR = 3.31 (1.83, 6.00) *P* < 0.0001), when the values were used as three-category variables and as a trend variable was also positively correlated with each mortality rate. Especially as the categorical variables, a dose-response relationship was clearly observed, that was, as the category of RAR increased (Q1 to Q3), the HR value of the risk of death gradually steadily increased. Such a relationship can also be observed in the external validation database. In the subgroup analysis, we observed an increased risk of death in the patient with hyperlipidemia and low HAS-BLED scores; however, no significant interaction was found in other subgroup analyses (including the diagnostic sequence of AIS).

**Conclusion:**

RAR was a predictor of mortality in AIS patients. However, more in-depth research is needed to further analyze and confirm the role of RAR in AIS patients.

## 1. Background

The global incidence of stroke was 17 million per year [1]. Acute ischemic stroke (AIS) was the leading cause of death and permanent disability [2]. Among the 50 million stroke survivors worldwide, 25% to 74% needed some help or completely depended on caregivers for activities of daily living (ADL) after stroke [3]. In China, due to the deepening of aging and risk factors, the burden of stroke continues to increase. A data [4] from 232 hospitals in China shows that although the severity of stroke patients in China is lower than that in other countries, but the patients in China are younger, therefore, we need to be more vigilant to identify the corresponding risk factors in advance. The Chinese Ministry of Health Stroke Prevention Project Committee (CSPPC) has established 380 stroke centers in China [5], including our hospital, and established a network of stroke centers, a stroke map, and a “green channel” for stroke. The CSPPC has monitored the quality of stroke care in stroke center hospitals through the China Stroke Data Center data reporting platform. The CSPPC Stroke program has led to a significant improvement in stroke care.

Therefore, identifying the markers of its risk factors was particularly important for screening high-risk groups, accurately predicting the outcome, formulating appropriate treatment goals, and selecting appropriate management strategies [6]. Research results in recent years had shown that the red cell distribution width (RDW) levels of blood [7] and human serum albumin [8] (ALB) are both predictors of the mortality risk of cerebrovascular disease. RDW was a simple parameter in the blood routine and an indicator that reflects the heterogeneity of the red blood cell size. It was often expressed by the coefficient of variation of the red blood cell volume [9]. ALB was the most abundant water-soluble protein in plasma. It was synthesized in the liver and has only one spherical polypeptide chain composed of 585 amino acid residues. It played an important role in maintaining plasma colloidal osmotic pressure and body nutritional balance [10]. Another report believed that the ratio of RDW to ALB (RAR) was a combined new indicator that can predict the prognosis of cardiovascular and respiratory diseases [11, 12]. However, it has not been effectively evaluated in cerebrovascular disease. This study will explore the correlation between RAR of first admission time and the 30-day all-cause mortality of 1412 AIS patients, in order to provide some evidences for the clinical prevention, treatment, and prognosis of AIS patients.

## 2. Materials and Methods

### 2.1. Data Source

The data were collected from a large US-based critical care database called Medical Information Mart for Intensive Care- (MIMIC-) IV database (version:1.0) [13]. The author Ping Liu had gained access to the MIMIC database (record ID: 37719988). Authors Quan Zhou and Xiangjie Duan used the PostgreSQL tool (version 9.6) to extract relevant data. Collect patients' general information (age, gender, race, and whether they drink alcohol), comorbidities (hypertension, hyperlipidemia, diabetes, coronary heart disease, atrial fibrillation, chronic lung disease, congestive heart failure, dementia, connective tissue disease, peripheral vascular disease, peptic ulcer, liver disease, malignant tumor, and HIV), vital signs (heart rate, blood pressure, respiration, body temperature, and SPO2) of the first admission time, laboratory data (blood routine, liver and kidney function, electrolytes, and coagulation function), various scores (APSIII, SAPSII, SOFA, Charlson Comorbidity Index SIRS score, and HAS-BLED score), medication (secondary prevention drugs for AIS include antiplatelet agents and anticoagulants included warfarin and other new anticoagulants), and special treatment (percutaneous endoscopic stomach and enterostomy) conditions. The primary endpoint was 30-day all-cause mortality, and the secondary outcome was ICU mortality and hospital mortality. Survival information was obtained from a table named “patients” in the MIMIC-IV database, and hospital stay data was extracted from a table named “admissions” in the MIMIC-IV database. Data of out-of-hospital deaths were obtained from the MIMIC-IV2.0 database.

### 2.2. Selection Criteria and Process

**Figure 1:**
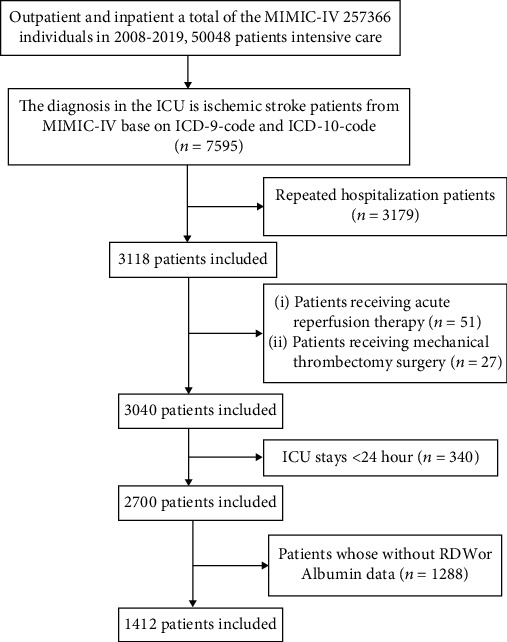
Research objection selection process.

The patients in Beth Israel Deaconess Medical Center (BIDMC) from 2008 to 2019 were identified in the MIMIC-IV database. The diagnostic criteria for AIS in this study were as follows: ICD-10: I63 and ICD-9: 34660, 34661, 34662, 34663, 43301, 43311, 43321, 43331, 43381, 43391, 43401, 43411, and 43491. This dataset excludes the following populations: (1) people younger than 18 years old, (2) patients receiving acute reperfusion therapy, and (3) patients receiving mechanical thrombectomy surgery. For patients who had been admitted to the ICU multiple times, we only use the data from the first admission. In addition, those who lacked relevant primary data (such as RDW and albumin) were also excluded. The specific selection process was shown in Figure 1.

### 2.3. RAR Measurement

Venous blood samples were taken from the patients within 24 hours after admission. RAR was calculated as the ratio of RDW to ALB. In order to reveal the exact relationship between these hematology parameters and the endpoints, we treated RAR as continuous variables, three categorical variables, and trend variables. In addition, due to the lack of repeated measures data of ALB, we collected and analyzed the repeated measures data of RDW.

### 2.4. Data Sources and Selection Criteria and Process for the Validation Cohort

We collected data from the eICU Collaborative Research Database (eICU-CRD) v2.0. The eICU-CRD was a multicenter ICU database which is maintained by the Laboratory for Computational Physiology at the Massachusetts Institute of Technology. The database contains health data for 200859 ICU admissions out of 139367 patients that stay at 208 United States hospitals from 2014 to 2015 [14]. They included hourly physiological readings from bedside monitors, records of demographics, severity of illness measures, medical history, clinical data, laboratory data, diagnoses via the Ninth Revision of International Classification of Diseases (ICD-9) codes, and other clinical data, collected during routine medical care. The primary outcome was all-cause in-hospital mortality. We found all patients with a diagnosis of AIS and then removed those who lacked RAR data.

### 2.5. Statistical Analysis

The baseline characteristics of all patients were stratified according to three categories of log-transformed RAR values. Categorical variables were described by frequency and percentage. Continuous variables that obey the normal distribution were described by mean ± standard; continuous variables which do not obey the normal distribution were described by the median (interquartile range (IQR)). We used univariate analysis and multivariate logistic regression model to evaluate whether RAR was independently associated with mortality in AIS patients. The results were expressed by odds ratios (ORs) and 95% confidence intervals (CI), after univariate analysis variables confirmed to have an impact on the results were used as covariates (*P* < 0.05) and adjusted in the multivariate logistic regression model. Based on clinical experience or literature, other variables that may affect RAR or outcome were also introduced as covariates. Since the RAR values were obviously non-normally distributed, we log transformed the RAR values. We used a multivariate logistic regression model analysis to test the stability of the results, and the log-transformed RAR values were used as continuous variables, three categorical variables, and trend variables to verify the result. We used the same approach to validate our inferences in the external validation cohort, and repeated measures data for RDWD were analyzed using a linear mixed-effects regression model. At last, we used a stratified logistic regression model to analyze whether the effects of the log-transformed RAR values on different subgroups were the same (including the diagnostic sequence of AIS, age, gender, hyperlipidemia, and atrial fibrillation). The two-sided probability value *P* < 5% was considered to be statistically significant, and all reported *P* values were two sided.

## 3. Result

### 3.1. Patient Characteristics

A total of 1412 patients with AIS were included in this study.  Table 1 summarized the baseline characteristics of patients according to the three groups of log-transformed RAR values. The categorical variables were expressed by the number of cases (percentage), and the continuous variables were normally distributed after the Pearson chi-square normality test and expressed by the mean ± standard deviation. The average age of the patients was 68.8 ± 15.9, including 708 (50.1%) men. According to different log-transformed RAR values, they were divided into three groups, named Q1 (0.8–1.2, *N* = 471), Q2 (1.2–1.5, *N* = 469), and Q3 (1.5–2.7, *N* = 472). Patients with higher RAR values were more likely to report a history of hyperlipidemia, atrial fibrillation, myocardial infarction, congestive heart failure, dementia, chronic pulmonary disease, rheumatic disease, peptic ulcer disease, diabetes, paraplegia, kidney disease, cancer, liver disease, and hypertension. Patients in the higher RAR group had a higher APSIII, SAPSII, Charlson Comorbidity Index, SIRS and SOFA score, heart rate, respiration rate, BUN, creatinine, WBC, chlorine, potassium, PT, APTT, ALT, ALP, AST, ICU mortality, 30-day all-cause mortality, and hospital mortality and had a lower MBP, bicarbonates, hematocrit, hemoglobin, MCH, MCHC, and RBC compared with those in the lower RAR group.

The data of RDW and ALB in this study were the values within 24 hours after the first admission, and the missing data were treated as missing percentages: variables with more than 5% missing values were excluded from the analysis; there were 1288 cases of missing values of ALB within 24 hours after the first admission, so we deleted this part of the population, but we added a sensitivity analysis as shown in Supplementary table 1.

The validation cohort included 2620 patients, including 2226 (84.96%) survivors before hospital discharge. The study patients had an average age of 67.51 ± 14.96 years and 1355 (51.72%) patients were male. The median with min-max of the log-transformed RAR level was 1.46 (0.97–2.69).

### 3.2. The Relationship between RAR and the Mortality

We used univariate and multivariate analysis showed in Table 2 to express the odds ratio (OR) and 95% confidence interval (CI) between log-transformed RAR values and 30-day all-cause mortality, ICU mortality, and hospital mortality. In the analysis, the group with the lower log-transformed RAR values was used as the baseline reference for comparison of the other types of groups. We found that when RAR were used as continuous variables, as the value increases, the risk of death increases (30-day all-cause mortality OR = 4.02(2.21, 7.32) < 0.0001, ICU mortality OR = 3.81 (1.92, 7.54), and hospital mortality OR = 3.31 (1.83, 6.00)), when RAR were used as three-category variables and trend variables were also positively correlated with each mortality rate. Especially as categorical variables, a dose-response relationship was clearly observed; when the subcategory increased, the HR value of the risk of each death gradually increased steadily.

It is indeed necessary to compare the predictive power of RAR indicators and other inflammatory biomarkers in terms of predictive efficacy. Therefore, we chose WBC, which is also an inflammatory marker, to compare with RAR. Comparing the diagnostic performance of WBC and RAR in predicting 30-day mortality, the ROC curve of the prediction model was analyzed as shown in Supplement Figure 1.

### 3.3. Relationship between RAR and Hospital Mortality in the Validation Cohort

We also used a multivariate logistic regression model on the validation database. We found in the results shown in Table 3 that in the validation cohort, as the RAR increased, the risk of death increased; whether the RAR was observed as a continuous variable, as a three-category variable, or as a trend variable, a dose-response relationship was also clearly observed when observed as categorical variables.

### 3.4. RDW Repeated Measures Generalized a Linear Mixed-Effects Regression Model analysis

**Figure 2:**
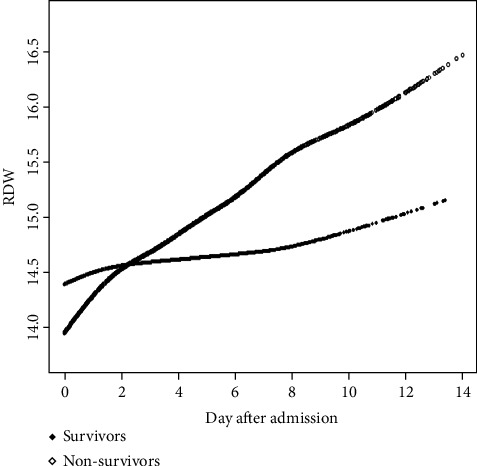
Association between changes in RDW and mortality.

The results were shown in Figure 2 and Table 4. It can be seen in Figure 1 that at both death group and the survival group, the value of log-transformed RDW had a linear upward trend with the increase in hospitalization time. The rising slope of the death group was greater than that of the survival group; it can be seen in Table 4 that the log-transformed RDW value of the death group increased by 1.0052 per day more than that of the survival group.

### 3.5. Subgroup Analysis

Subgroup analysis of the association between the log-transformed RAR values and 30-day all-cause mortality was performed (Table 5). We observed an increased risk of death in the patient with hyperlipidemia OR (95%CI) = 10.08 (3.91, 25.96), *P* for interaction = 0.0055 and low HAS-BLED scores HR (95%CI) = 5.53 (2.87, 10.66), *P* for interaction < 0.0003; however, no significant interaction was found in other subgroup analyses (including the diagnostic sequence of AIS).

## 4. Discussion

In this paper, a large retrospective cohort study of AIS patients showed that the log-transformed RAR values were significantly positively correlated with 30-day all-cause mortality, ICU mortality, and hospital mortality, even after adjusting for age, gender, race, and other correlations. Patients with higher log-transformed RAR values were more likely to have a poorer clinical prognosis and higher mortality both as a continuous variable, as a categorical variable, and as a trend variable. Moreover, this correlation was also clearly observed in the validation cohort and repeated measures analysis. Therefore, a new, cheap, commonly used, and easily available clinical index can be easily used by doctors to assess the prognosis of AIS patients.

In blood test results, RDW reflects the dispersion of the size of peripheral red blood cells. The increasing of the RDW level indicates that the red blood cell volume is more dispersed in peripheral blood. Therefore, it is often used together with other blood cell parameters to identify blood system diseases such as anemia [15]. Previous literature studies have shown that AIS patients had higher RDW in the population with poor postoperative functional outcome [16], so we excluded 27 patients who underwent mechanical thrombectomy, although the important role of reperfusion therapy in the treatment of acute myocardial infarction has been well documented. However, reperfusion therapy can trigger an inflammatory response and possibly damage the myocardium, leading to poor outcomes [17], so we deleted 51 patients who received acute reperfusion therapy. The RDW may be related to certain physiological processes [18] and pathological processes [19]; RDW may be a biomarker reflecting the state of the body; a sample size of 15852 researches on adults in the community had shown that higher RDW was closely related to the risk of all-cause death [20]. Studies had proposed that the RDW value before intravenous thrombolysis was an independent predictor of mortality in patients with AIS [21], and a study of patients with coronary heart disease showed that the RDW level was high significantly correlated with the increase of the incidence of stroke [22]. In addition, other previous studies had also reported that RDW may provide prognostic information for the function of stroke patients [23, 24]. These conditions were the result of a variety of mechanisms, including inflammation, dyslipidemia, and oxidative stress. First, the blood lipid content in the red blood cell membrane is very important to maintain the stability of the red blood cell. Excessive increase of the cholesterol content in the membrane will reduce the flowing of the red blood cell membrane, making it prone to rupture [25]. The deposition of free cholesterol in the blood vessel wall and the rupture and accumulation of red blood cells can easily lead to atherosclerosis, increase the volume of the necrotic lipid core, accelerate the rupture of atherosclerotic plaque, and induce acute thrombotic events [26]. Therefore, that maybe the reason that we found in subgroup analysis that there is a significant positive correlation between hyperlipidemia patients and mortality HR (CI) = 1.42 (1.26, 1.59), but it was not found in nonhyperlipidemia patients. Secondly, inflammation is a key issue in atherosclerosis and ischemic stroke [27, 28]; increased peripheral blood RDW was related to inflammation [29]. Inflammatory mediators can inhibit the production and utilization of EPO; hinder the absorption, transportation, and utilization of iron; reduce the iron concentration in blood circulation; and at last hinder the production and maturation of red blood cells [30]. In addition, oxidative reaction can accelerate the aging and rupture of red blood cells, thereby changing the ratio between cell subpopulations and volume distribution [31]. Therefore, the increase of RDW may be due to the promotion of aging and rupture of red blood cells and the release of immature red blood cells. These mechanisms influence and interact with each other caused by entering the peripheral blood circulation and jointly mediate the significant correlation between RDW and the mortality of AIS patients.

ALB is the most abundant protein in plasma. It has many biological functions, including maintaining plasma colloidal osmotic pressure and combining and transporting endogenous and exogenous substances and drugs; it can reflect the nutritional status of the body [32], has antioxidant, anti-inflammatory [33], antiaggregation, and anticoagulant effects [34], and can also be used as a biomarker for many human acute and chronic diseases [35]. A survey of healthy people found that a relatively low ALB level has a higher risk of cardiovascular disease [36]. Hashem [37] found that ALB was an important prognostic indicator after AIS. Through linear regression analysis, they found that ALB was the only important predictor in their research. A study from China using a nomogram chart prediction model found that ALB was one of the predictors of death within 6 months of stroke onset (OR = 0.854, 95% CI = 0.774 − 0.931, *P* < 0.01). A number of studies had shown that high ALB levels were associated with better prognosis in stroke patients [38–40]. The relationship between ALB and the death and prognosis of stroke patients may occur through the following mechanisms. The first mechanism was due to the fact that ALB contains a free cysteine residue that played an important redox affecting outside the cell [33, 41], which can selectively inhibit the expression of TNF-*α*-induced vascular cell adhesion molecules and enhance the adhesion of monocytes and activation of kappa-B [42]. The second mechanism was that ALB can bind to arachidonic acid, inhibit the production and activity of thromboxane A2, and induce macrophages producing enzymes to promote the formation of nitric oxide, thereby inhibiting the aggregation of platelet, and can also bind with prostacyclin 12 to enhance its antiplatelet aggregation effect. In addition, ALB also had an antithrombin effect similar to heparin [34]. In the subgroup analysis of this study, among the people with low HAS-BLED (<3) bleeding tendency score, when the RAR value increases, the reason maybe was the decreasing of ALB and the body's antiplatelet aggregation and antithrombin effects were reduced, which made it easier to form plaques, induce acute thrombosis, and at last lead to an increasing in mortality HR (95%CI) = 1.18 (1.10, 1.28), which was meaningless in patients with high HAS-BLED (≥3). The third mechanism was that ALB stimulated immune cells to exert neuroprotective effect. By maintaining the integrity of the blood-brain barrier, it reduced brain edema and reduced apoptosis and inflammation [8]. When stroke patients were accompanied by hypoproteinemia, it will aggravate the inflammation in the body, reduce the elimination of oxygen free radicals, and make the blood system be in a hypercoagulable state. These were all related to a poor prognosis. Therefore, relatively low ALB levels may be a risk factor.

Although the above studies all support that RDW and ALB had a predictive value for cerebrovascular diseases, they lack specificity and standardization and had not been applied in clinical practice. This study combines them to form a new indicator RAR, which may enhance the capability of its prediction. Especially a dose-response relationship was clearly observed; when the RAR subcategory increased, the HR value of the risk of each death gradually increased steadily.

## 5. Advantages

This study was a retrospective observational study with a large sample size. It used logistic regression models to analyze and conducted a stratified analysis interaction test. Finally, we found a dose-response relationship that the RAR level can stably predict 30-day mortality, ICU mortality, and in-current hospital mortality.

We used an external database to verify the results of the study and performed repeated measures analysis on one of the combined indicators (RDW), which all can well support the results of this study.

RAR had some clinical advantages: On the one hand, it had the advantages of simplicity, speed, and low cost. It does not require special skills and inspection equipment for monitoring. On the other hand, almost all medical institutions, including grassroot community hospitals, had the routine monitored.

## 6. Limitations of This Study

Potential confounding factors were inevitable. Therefore, we combine the existing literature, clinical judgment, and statistical methods to adjust confounding factors as much as possible but our results may still be affected by other unknown factors.

The levels of RDW and albumin were tested only once within 24 hours after admission; the dynamic changes of the combined indicators during hospitalization could not be analyzed. This should be considered in future studies to further verify the correlation between RAR and the prognosis of AIS patients.

This study was a single-center retrospective study, so the representativeness of the sample had certain limitations. It only applies to patients without thrombolysis or mechanical thrombectomy in AIS patients. Multicenter registration and prospective studies are needed to confirm this finding.

## 7. Conclusions

RAR is an independent predictor of hospital mortality in AIS patients. Furthermore, there is a dose-response relationship between RAR and hospital mortality.

## Figures and Tables

**Table 1:** Characteristics of the study patients.

Characteristics		RAR in transform groups (mL/g)					
		Q1 (*N* = 471) 0.8–1.2	Q2 (*N* = 469) 1.2–1.5	Q3 (*N* = 472) 1.5–2.7	*P* value	
General information	Sex, male, *N*	216 (45.9%)	241 (51.4%)	248 (52.5%)	0.090
	Age, mean, years	66.1 ± 15.6	71.6 ± 15.5	68.5 ± 16.2	<0.001
	Race, *N*				0.176
	White	288 (61.1%)	292 (62.3%)	280 (59.3%)	
	Black	46 (9.8%)	41 (8.7%)	67 (14.2%)	
	Asian	16 (3.4%)	12 (2.6%)	13 (2.8%)	
	Other	121 (25.7%)	124 (26.4%)	112 (23.7%)	
	Alcohol	2 (0.42%)	0 (0.00%)	7 (1.48%)	0.013

Comorbidities, *N*	Hyperlipidemia	271 (57.54%)	241 (51.39%)	196 (41.53%)	<0.001
	Atrial fibrillation	139 (29.51%)	191 (40.72%)	206 (43.64%)	<0.001
	Myocardial infarction	50 (10.62%)	76 (16.20%)	119 (25.21%)	<0.001
	Congestive heart failure	52 (11.04%)	121 (25.80%)	177 (37.50%)	<0.001
	Peripheral vascular disease	37 (7.86%)	47 (10.02%)	59 (12.50%)	0.061
	Dementia	14 (2.97%)	33 (7.04%)	27 (5.72%)	0.017
	Chronic pulmonary disease	49 (10.40%)	81 (17.27%)	96 (20.34%)	<0.001
	Rheumatic disease	6 (1.27%)	17 (3.62%)	25 (5.30%)	0.003
	Peptic ulcer disease	2 (0.42%)	7 (1.49%)	18 (3.81%)	<0.001
	Diabetes	137(14.54%)	173(18.44%)	203(21.50%)	<0.001
	Paraplegia	261 (55.41%)	255 (54.37%)	181 (38.35%)	<0.001
	Kidney disease	43 (9.13%)	85 (18.12%)	136 (28.81%)	<0.001
	Malignant tumor	15 (3.18%)	41 (8.74%)	75 (15.89%)	<0.001
	Metastatic cancer	4 (0.85%)	17 (3.62%)	38 (8.05%)	<0.001
	AIDS	1 (0.21%)	0 (0.00%)	3 (0.64%)	0.332
	Liver disease	6 (1.27%)	12 (2.56%)	54 (11.44%)	<0.001
	Hypertension	287 (60.93%)	245 (52.24%)	189 (40.04%)	<0.001

Score system	APSIII	38.49 ± 19.15	47.13 ± 21.97	65.53 ± 29.73	<0.001
	SAPSII	28.76 ± 10.39	35.23 ± 12.25	42.90 ± 14.71	<0.001
	HAS-BLED score	1.40 ± 0.96	1.51 ± 0.94	1.51 ± 0.94	0.122
	Charlson comorbidity index	6.09 ± 2.30	7.30 ± 2.54	7.99 ± 3.04	<0.001
	SIRS score	2.14 ± 0.98	2.35 ± 1.02	2.70 ± 0.94	<0.001
	SOFA	3.14 ± 2.56	4.58 ± 3.31	7.49 ± 4.73	<0.001

Vital signs	Heart rate (beat/minute)	80.69 ± 16.30	83.19 ± 18.58	92.15 ± 21.13	<0.001
	Respiration rate (breath/minute)	18.59 ± 4.75	19.40 ± 5.07	21.00 ± 6.30	<0.001
	MBP (mmHg)	98.39 ± 18.09	94.83 ± 18.96	87.01 ± 19.50	<0.001
	Temperature (°C)	36.78 ± 0.57	36.76 ± 0.71	36.79 ± 1.00	0.813
	SPO2 (%)	97.48 ± 2.73	97.24 ± 2.94	96.74 ± 4.37	0.003

Laboratory results	BUN (mg/dL)	18.47 ± 9.66	22.15 ± 13.30	33.09 ± 23.70	<0.001
	Creatinine (mEq/L)	1.06 ± 0.91	1.23 ± 1.09	1.84 ± 2.02	<0.001
	WBC (10^9^/L)	10.93 ± 4.81	11.35 ± 5.78	12.34 ± 7.23	0.001
	Bicarbonates (mmol/L)	22.93 ± 3.43	22.57 ± 3.87	21.43 ± 5.17	<0.001
	Anion gap (mmol/L)	16.09 ± 4.08	15.74 ± 4.05	16.46 ± 5.58	0.062
	Chlorine (mmol/L)	102.41 ± 5.15	102.88 ± 5.57	103.57 ± 7.30	0.013
	Blood sugar (mg/dL)	144.51 ± 69.27	156.74 ± 98.42	159.17 ± 113.81	0.043
	Sodium (mmol/L)	139.17 ± 4.27	138.71 ± 4.70	138.72 ± 5.59	0.259
	Potassium (mmol/L)	4.24 ± 0.90	4.27 ± 0.78	4.38 ± 0.96	0.038
	PT	12.61 ± 3.94	13.92 ± 5.29	16.06 ± 7.13	<0.001
	Platelets	232.06 ± 74.18	224.77 ± 89.81	216.04 ± 132.54	0.055
	APTT	30.99 ± 16.16	32.45 ± 16.74	34.77 ± 17.96	0.003
	Hematocrit	41.14 ± 4.50	37.87 ± 5.51	32.26 ± 7.37	<0.001
	Hemoglobin	13.72 ± 1.57	12.44 ± 1.86	10.35 ± 2.46	<0.001
	MCH	30.52 ± 2.02	29.95 ± 2.31	29.10 ± 3.18	<0.001
	MCHC	33.37 ± 1.32	32.86 ± 1.49	32.05 ± 1.85	<0.001
	MCV	91.54 ± 5.65	91.22 ± 6.35	90.84 ± 8.63	0.315
	RBC	4.51 ± 0.57	4.17 ± 0.65	3.59 ± 0.88	<0.001
	ALT	35.84 ± 100.50	50.37 ± 176.33	139.25 ± 474.68	<0.001
	ALP	81.06 ± 49.47	89.16 ± 50.47	109.09 ± 83.19	<0.001
	AST	48.59 ± 144.26	83.31 ± 365.07	202.62 ± 671.75	<0.001

Treatment, *N*	Warfarin	86 (18.26%)	86 (18.34%)	108 (22.88%)	0.125
	NOAC	67 (14.23%)	77 (16.42%)	51 (10.81%)	0.042
	ANTIPLT	362 (76.86%)	357 (76.12%)	309 (65.47%)	<0.001
	PEG/PEJ	19 (4.03%)	25 (5.33%)	29 (6.14%)	0.337

Death situation, *N*	30-day all-cause mortality	60 (12.74%)	82 (17.48%)	142 (30.08%)	<0.001
	ICU mortality	42 (8.92%)	49 (10.45%)	84 (17.80%)	<0.001
	In-current hospital mortality	59 (12.53%)	83 (17.70%)	141 (29.87%)	<0.001

APSIII: acute physiology score III; SAPSII: simplified acute physiology score II; HAS-BLED: has-bled bleeding risk score; SIRS: system inflammatory response syndrome; SOFA: sequential organ failure assessment; MBP: mean blood pressure; BUN: blood urea nitrogen; WBC: white blood cell; PT: prothrombin time; APTT: activated thrombin time; MCH: average hemoglobin content; MCHC: average hemoglobin concentration; MCV: average red blood cell volume; RBC: total red blood cell; ALT: alanine transaminase; ALP: alkaline phosphatase; AST: aspartate aminotransferase; NOAC: new oral anticoagulant; ANTIPLT: antiplatelet drugs; PEG/PEJ: percutaneous endoscopic gastrostomy/jejunostomy.

**Table 2:** Association between different RAR in transform levels and outcomes among AIS patients.

Outcomes		Nonadjusted model OR (95% CI) *P* value	Minimally adjusted model OR (95% CI) *P* value	Fully adjusted model OR (95% CI) *P* value
30-day all-cause mortality	RAR in transform	4.50 (2.93, 6.91) <0.0001	4.89 (3.12, 7.66) <0.0001	4.02 (2.21, 7.32) <0.0001
	RAR (three groups)			
	Q1	Ref	Ref	Ref
	Q2	1.45 (1.01, 2.08) 0.0430	1.34 (0.92, 1.94) 0.1224	1.64 (1.13, 2.37) 0.0085
	Q3	2.95 (2.11, 4.12) <0.0001	3.03 (2.15, 4.27) <0.0001	2.75 (1.81, 4.19) <0.0001
	*P* for trend	1.75 (1.48, 2.07) <0.0001	1.79 (1.50, 2.13) <0.0001	1.66 (1.35, 2.05) <0.0001

ICU mortality	RAR in transform	3.58 (2.18, 5.87) <0.0001	3.59 (2.17, 5.95) <0.0001	3.81 (1.92, 7.54) 0.0001
	RAR (three groups)			
	Ref	Ref	Ref	Ref
	Q2	1.19 (0.77, 1.84) 0.4279	1.15 (0.74, 1.79) 0.5262	1.23 (0.78, 1.95) 0.3779
	Q3	2.21 (1.49, 3.28) <0.0001	2.20 (1.48, 3.29) 0.0001	1.96 (1.18, 3.25) 0.0093
	*P* for trend	1.52 (1.24, 1.86) <0.0001	1.52 (1.24, 1.87) <0.0001	1.40 (1.09, 1.81) 0.0092

Hospital mortality	RAR in transform	4.70 (3.06, 7.21) <0.0001	5.09 (3.25, 7.98) <0.0001	3.31 (1.83, 6.00) <0.0001
	RAR (three groups)			
	Q1	Ref	Ref	Ref
	Q2	1.50 (1.05, 2.16) 0.0275	1.40 (0.97, 2.03) 0.0758	1.47 (1.00, 2.17) 0.0492
	Q3	2.97 (2.12, 4.16) <0.0001	3.07 (2.17, 4.33) <0.0001	2.15 (1.39, 3.31) 0.0005
	*P* for trend	1.75 (1.48, 2.07) <0.0001	1.79 (1.51, 2.14) <0.0001	1.47 (1.18, 1.82) 0.0005

Nonadjusted model adjusts for the following: no covariates were adjusted for. Minimally adjusted model: we only adjusted for sex, ethnicity, and age. Fully adjusted model: we adjusted for age, sex, ethnicity, heart rate, BUN, anion gap, hematocrit, NOAC, and ANTIPLT; PEG/PEJ. BUN: blood urea nitrogen; NOAC: new oral anticoagulant; ANTIPLT: antiplatelet drugs; PEG/PEJ: percutaneous endoscopic gastrostomy/jejunostomy; Ref: reference.

**Table 3:** Association between different RAR levels and outcomes among AIS patients in the validation database.

Validation EICU hospital mortality	Nonadjusted model OR (95% CI) *P* value	Minimally adjusted model OR (95% CI) *P* value	Fully adjusted model OR (95% CI) *P* value
RAR	1.3 (1.2, 1.4) < 0.001	1.3 (1.2, 1.4) < 0.001	1.2 (1.0, 1.3) 0.010
RAR (three groups)			
Q1	1.0	1.0	1.0
Q2	1.1 (0.8, 1.5) 0.384	1.1 (0.8, 1.5) 0.484	1.1 (0.7, 1.7) 0.687
Q3	2.4 (1.8, 3.1) < 0.001	2.4 (1.8, 3.2) < 0.001	1.6 (1.1, 2.5) 0.024
*P* for trend	1.6 (1.4, 1.8) < 0.001	1.6 (1.4, 1.8) < 0.001	1.3 (1.0, 1.6) 0.016

Nonadjusted model adjusts for the following: no covariates were adjusted for. Minimally adjusted model: we only adjusted for sex, ethnicity, and age. Adjust II model adjusts for the following: sex, ethnicity, age, GCS, hypertension, angina; cardiovascular disease, cirrhosis, COPD, diabetes, stroke, TIA, BUN, anion gap, and WBC. GCS: Glasgow Coma Scale; COPD: chronic obstructive pulmonary disease; TIA: transient ischemia attack; BUN: blood urea nitrogen; WBC: white blood cell.

**Table 4:** Predictors of longitudinal log-transformed RDW derived from a linear mixed-effects regression model.

Variable	Coefficient	Standardized error	95% CI	*P* value
Intercept	14.0871	0.0512	13.9868-14.1874	<0.0001
Time	0.1132	0.0033	0.1066-0.1197	<0.0001
Time × death	1.0052	0.1081	0.7933-1.2170	<0.0001

CI: confidence interval; intercept: the mean of log-transformed RDW count at day = 0 and death = 0; time: the mean of the increasing of log-transformed RDW count at death = 0 over time(daily); time × death: the average increasing in log-transformed RDW count daily under the condition of the group of death = 1 compared with the group of death = 0.

**Table 5:** Subgroup analysis of the relationship between RAR in transform and 30-day hospital mortality.

Characteristic	Number of patients	OR (95% CI)	*P* value	*P* for interaction
Diagnosis sequence				0.4760
1	746	4.5 (1.7, 11.7)	0.0023	
2	227	5.1 (1.1, 23.8)	0.0375	
≥3	429	2.2 (0.9, 5.3)	0.0799	
Age (years)				0.5838
<65	529	4.55 (1.61, 12.81)	0.0041	
≥65	873	3.23 (1.57, 6.63)	0.0014	
Sex				0.4686
Female	700	4.38 (2.04, 9.40)	0.0001	
Male	702	3.11 (1.45, 6.66)	0.0036	
Hyperlipidemia				0.0055
No	700	1.95 (0.93, 4.09)	0.0792	
Yes	702	10.08 (3.91, 25.96)	<0.0001	
Atrial fibrillation				0.5183
No	869	4.35 (2.01, 9.41)	0.0002	
Yes	533	2.98 (1.21, 7.34)	0.0178	
Hypertension				0.4123
No	686	4.65 (2.04, 10.59)	0.0003	
Yes	716	2.89 (1.25, 6.68)	0.0132	
HAS-BLED				0.0003
<3	1243	5.53 (2.87, 10.66)	<0.0001	
≥3	159	0.14 (0.02, 1.04)	0.0543	
NOAC				0.3727
No	1209	3.42 (1.84, 6.33)	<0.0001	
Yes	193	13.39 (0.72, 249.11)	0.0819	

We adjusted for age, sex, ethnicity, heart rate, BUN, anion gap, hematocrit, NOAC, and ANTIPLT; PEG/PEJ. NOAC: new oral anticoagulant.

## Data Availability

The datasets are publicly available in https://mimic.physionet.org/.

## Supplementary Materials

Supplementary Materials The data of RDW and ALB in this study were the values within 24 hours after the first admission, and the missing data were treated as missing percentages: variables with more than 5% missing values were excluded from the analysis, there were 1288 cases of missing values of ALB within 24 hours after the first admission, so we deleted this part of the population, but we added a sensitivity analysis as shown as follows. Supplementary Figure 1: it is indeed necessary to compare the predictive power of RAR indicators and other inflammatory biomarkers in terms of predictive efficacy. Therefore, we chose WBC, which is also an inflammatory marker, to compare with RAR. Comparing the diagnostic performance of WBC and RAR in predicting 30-day mortality, the ROC curve of the prediction model was analyzed as shown as follows.

## References

[1] Feigin V. L., Forouzanfar M. H., Krishnamurthi R. (2014). Global and regional burden of stroke during 1990-2010: findings from the Global Burden of Disease Study 2010. *The Lancet*.

[2] Benjamin E. J., Muntner P., Alonso A. (2019). Heart disease and stroke statistics-2019 update: a report from the American Heart Association. *Circulation*.

[3] Miller E. L., Murray L., Richards L. (2010). Comprehensive overview of nursing and interdisciplinary rehabilitation care of the stroke patient. *Stroke*.

[4] Tu W. J., Chao B. H., Ma L. (2021). Case-fatality, disability and recurrence rates after first-ever stroke: a study from bigdata observatory platform for stroke of China. *Brain Research Bulletin*.

[5] Chao B. H., Yan F., Hua Y. (2021). Stroke prevention and control system in China: CSPPC-stroke program. *International Journal of Stroke*.

[6] Gong P., Liu Y., Gong Y. (2021). The association of neutrophil to lymphocyte ratio, platelet to lymphocyte ratio, and lymphocyte to monocyte ratio with post-thrombolysis early neurological outcomes in patients with acute ischemic stroke. *Journal of Neuroinflammation*.

[7] Mo L., Chen Y., Li Z. (2017). Red blood cell distribution width as a marker of cerebral infarction in hemodialysis patients. *Renal Failure*.

[8] Yang X., Wang L., Zheng L. (2020). Serum albumin as a potential predictor of pneumonia after an acute ischemic stroke. *Current Neurovascular Research*.

[9] Zöller B., Melander O., Svensson P., Engström G. (2014). Red cell distribution width and risk for venous thromboembolism: a population- based cohort study. *Thrombosis Research*.

[10] Jahanban-Esfahlan A., Ostadrahimi A., Jahanban-Esfahlan R., Roufegarinejad L., Tabibiazar M., Amarowicz R. (2019). Recent developments in the detection of bovine serum albumin. *International Journal of Biological Macromolecules*.

[11] Yoo J. W., Ju S., Lee S. J., Cho Y. J., Lee J. D., Kim H. C. (2020). Red cell distribution width/albumin ratio is associated with 60-day mortality in patients with acute respiratory distress syndrome. *Infectious Diseases*.

[12] Long J., Xie X., Xu D. (2021). Association between red blood cell distribution width-to-albumin ratio and prognosis of patients with aortic aneurysms. *International Journal of General Medicine*.

[13] Goldberger A. L., Amaral L. A., Glass L. (2000). PhysioBank, PhysioToolkit, and PhysioNet: components of a new research resource for complex physiologic signals. *Circulation*.

[14] Pollard T. J., Johnson A., Raffa J. D., Celi L. A., Mark R. G., Badawi O. (2018). The eICU collaborative research database, a freely available multi-center database for critical care research. *Scientific data*.

[15] Ćatić J., Jurin I., Lucijanić M., Jerkić H., Blažeković R. (2018). High red cell distribution width at the time of ST segment elevation myocardial infarction is better at predicting diastolic than systolic left ventricular dysfunction. *Medicine*.

[16] Akpinar C. K., Gurkaş E., Aykac O., Uysal Z., Ozdemir A. O. (2021). Elevated red blood cell distribution width may be a novel independent predictor of poor functional outcome in patients treated with mechanical thrombectomy. *Neurointervention*.

[17] Garjani A., Sohrabi B., Movassaghpour A. A. (2016). Thrombolytic therapy up-regulates inflammatory mediators compared to percutaneous coronary intervention (PCI). *Iranian Journal of Allergy, Asthma, and Immunology*.

[18] Paliogiannis P., Zinellu A., Mangoni A. A. (2018). Red blood cell distribution width in pregnancy: a systematic review. *Biochemia medica*.

[19] Qian H., Luo Z., Xiao C. (2018). Red cell distribution width in coronary heart disease: prediction of restenosis and its relationship with inflammatory markers and lipids. *Postgraduate Medical Journal*.

[20] Perlstein T. S., Weuve J., Pfeffer M. A., Beckman J. A. (2009). Red blood cell distribution width and mortality risk in a community-based prospective Cohort. *Archives of Internal Medicine*.

[21] Ye W. Y., Li J., Li X. (2020). Predicting the one-year prognosis and mortality of patients with acute ischemic stroke using red blood cell distribution width before intravenous thrombolysis. *Clinical Interventions in Aging*.

[22] Wu H., Wang X., Zhang J., Sun H. (2019). Can red blood cell distribution width predict long-term cardiovascular event after off-pump coronary artery bypass? A retrospective study. *Journal of Cardiac Surgery*.

[23] Turcato G., Cappellari M., Follador L. (2017). Red blood cell distribution width is an independent predictor of outcome in patients undergoing thrombolysis for ischemic stroke. *Seminars in Thrombosis and Hemostasis*.

[24] Kara H., Degirmenci S., Bayir A. (2015). Red cell distribution width and neurological scoring systems in acute stroke patients. *Neuropsychiatric Disease and Treatment*.

[25] De Freitas M. V., De Oliveira M. R., Dos Santos D. F., de Cássia Mascarenhas Netto R., Fenelon S. B., Penha-Silva N. (2010). Influence of the use of statin on the stability of erythrocyte membranes in multiple sclerosis. *The Journal of Membrane Biology*.

[26] Tziakas D., Chalikias G., Grapsa A., Gioka T., Tentes I., Konstantinides S. (2012). Red blood cell distribution width – a strong prognostic marker in cardiovascular disease – is associated with cholesterol content of erythrocyte membrane. *Clinical Hemorheology and Microcirculation*.

[27] Chamorro A., Dirnagl U., Urra X., Planas A. M. (2016). Neuroprotection in acute stroke: targeting excitotoxicity, oxidative and nitrosative stress, and inflammation. *Lancet Neurology*.

[28] Libby P., Loscalzo J., Ridker P. M. (2018). Inflammation, Immunity, and Infection in atherothrombosis:. *Journal of the American College of Cardiology*.

[29] Horta-Baas G., Romero-Figueroa M. (2019). Clinical utility of red blood cell distribution width in inflammatory and non- inflammatory joint diseases. *International Journal of Rheumatic Diseases*.

[30] Tanyong D. I., Panichob P., Kheansaard W., Fucharoen S. (2015). Effect of tumor necrosis factor-alpha on erythropoietin and erythropoietin receptor-induced erythroid progenitor cell proliferation in *β*-thalassemia/hemoglobin E Patients. *Turkish Journal of Haematology*.

[31] Mazzulla S., Schella A., Gabriele D. (2015). Oxidation of human red blood cells by a free radical initiator: effects on rheological properties. *Clinical Hemorheology and Microcirculation*.

[32] Kimura Y., Yamada M., Kakehi T., Itagaki A., Tanaka N., Muroh Y. (2017). Combination of low body mass index and low serum albumin level leads to poor functional recovery in stroke patients. *Journal of Stroke and Cerebrovascular Diseases*.

[33] Kawai Y., Masutani K., Torisu K. (2018). Association between serum albumin level and incidence of end-stage renal disease in patients with immunoglobulin a nephropathy: a possible role of albumin as an antioxidant agent. *PLoS One*.

[34] Paar M., Rossmann C., Nusshold C. (2017). Anticoagulant action of low, physiologic, and high albumin levels in whole blood. *PLoS One*.

[35] Al-Harthi S., Lachowicz J. I., Nowakowski M. E., Jaremko M., Jaremko Ł. (2019). Towards the functional high-resolution coordination chemistry of blood plasma human serum albumin. *Journal of Inorganic Biochemistry*.

[36] Ronit A., Kirkegaard-Klitbo D. M., Dohlmann T. L. (2020). Plasma albumin and incident cardiovascular disease. *Arteriosclerosis, Thrombosis, and Vascular Biology*.

[37] Hashem S. S., Helmy S. M., El-Fayomy N. M. (2018). Predictors of stroke outcome: the role of hemorheology, natural anticoagulants, and serum albumin. *The Egyptian journal of neurology, psychiatry and neurosurgery*.

[38] Dziedzic T., Slowik A., Szczudlik A. (2004). Serum albumin level as a predictor of ischemic stroke outcome. *Stroke*.

[39] Wang C., Deng L., Qiu S. (2019). Serum albumin is negatively associated with hemorrhagic transformation in acute ischemic stroke patients. *Cerebrovascular Diseases*.

[40] Idicula T. T., Waje-Andreassen U., Brogger J., Naess H., Thomassen L. (2009). Serum albumin in ischemic stroke patients: the higher the better. *Cerebrovascular Diseases*.

[41] González-Pacheco H., Amezcua-Guerra L. M., Sandoval J. (2017). Prognostic implications of serum albumin levels in patients with acute coronary syndromes. *The American Journal of Cardiology*.

[42] Celik I. E., Yarlioglues M., Kurtul A. (2016). Preprocedural albumin levels and risk of in-stent restenosis after coronary stenting with bare-metal stent. *Angiology*.

