# Supplementary figures and images for: RDW-to-ALB Ratio Is an Independent Predictor for 30-Day All-Cause Mortality in Patients with Acute Ischemic Stroke: A Retrospective Analysis from the MIMIC-IV Database
Source: Behav Neurol. 2022 Dec 15;2022:3979213. doi: 10.1155/2022/3979213 (PMC9780005; doi:10.1155/2022/3979213)

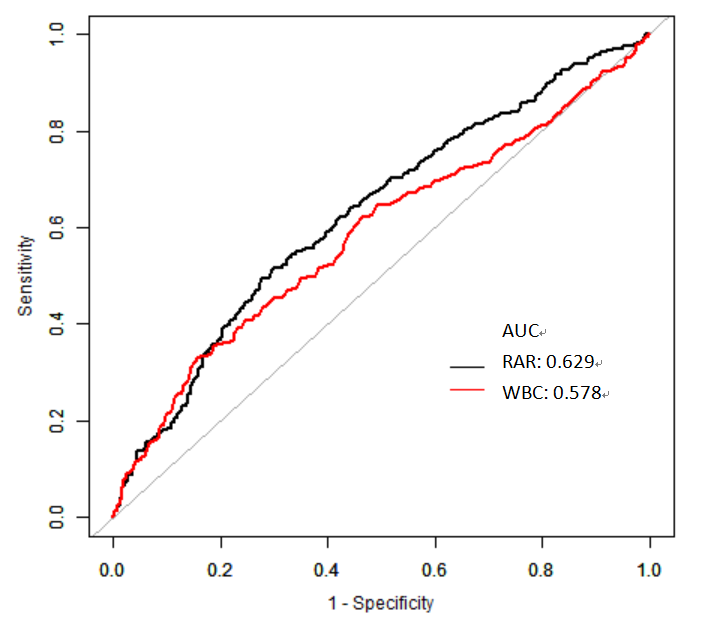


**Attachment Figure 1** **ROC curve of RAR and WBC**

Supplement: Supplementary Materials — The data of RDW and ALB in this study were the values within 24 hours after the first admission, and the missing data were treated as missing percentages: variables with more than 5% missing values were excluded from the analysis, there were 1288 cases of missing values of ALB within 24 hours after the first admission, so we deleted this part of the population, but we added a sensitivity analysis as shown as follows. Supplementary Figure 1: it is indeed necessary to compare the predictive power of RAR indicators and other inflammatory biomarkers in terms of predictive efficacy. Therefore, we chose WBC, which is also an inflammatory marker, to compare with RAR. Comparing the diagnostic performance of WBC and RAR in predicting 30-day mortality, the ROC curve of the prediction model was analyzed as shown as follows. [file 3979213.f1.zip › Supplementary Figure 1.docx]
